# Beverly Murphy, AHIP, FMLA, Medical Library Association President, 2018–2019

**DOI:** 10.5195/jmla.2018.575

**Published:** 2018-10-01

**Authors:** Shannon D. Jones

**Affiliations:** Director of Libraries, Medical University of South Carolina, Charleston, SC

## Abstract

When I think of Beverly Murphy, AHIP, FMLA, three words come to mind: Bold, Brave, Brilliant. In every action that Beverly has undertaken as a health sciences librarian, she has demonstrated an innate ability to just “Be.” Being bold, brave, and brilliant is just what she will need to be as the Medical Library Association’s first African-American president since the association’s founding in 1898.

Beverly Murphy, AHIP, FMLA, began her career as the technical services librarian for the University of Virginia’s Science/Technology Information Center in 1981. In 1983, she moved back to Durham to work as assistant librarian in the library of the *Herald-Sun Newspaper.* For thirty-five years, Beverly has worked for Duke University, where she serves as the assistant director for communications and web content management at the Duke University Medical Center Library and Archives and the hospital nursing liaison for the Duke Health System and Watts School of Nursing. As the liaison, she works with nursing faculty, staff, and students—teaching and assisting with their research needs for publication, quality improvement, performance evaluation, graduate residency, and class assignments. Other roles she has held at Duke include head, Reference Service; reference librarian; and user education coordinator.

A native North Carolinian, Beverly is a proud alumnus of North Carolina Central University (NCCU) in Durham, North Carolina, where she received a bachelor of science in biology and a master of science in library science.

Beverly is a long-time member of the Medical Library Association (MLA) and has provided meritorious service to the association. Since joining MLA in 1985, Beverly has served as president-elect, *MLA News* editor, a member of the Board of Directors, and a member of the *Journal of the Medical Library Association (JMLA)* Editorial Board. In 2015, she was awarded the status of fellow in recognition of her outstanding contributions to the health sciences librarianship profession.

**Figure:**
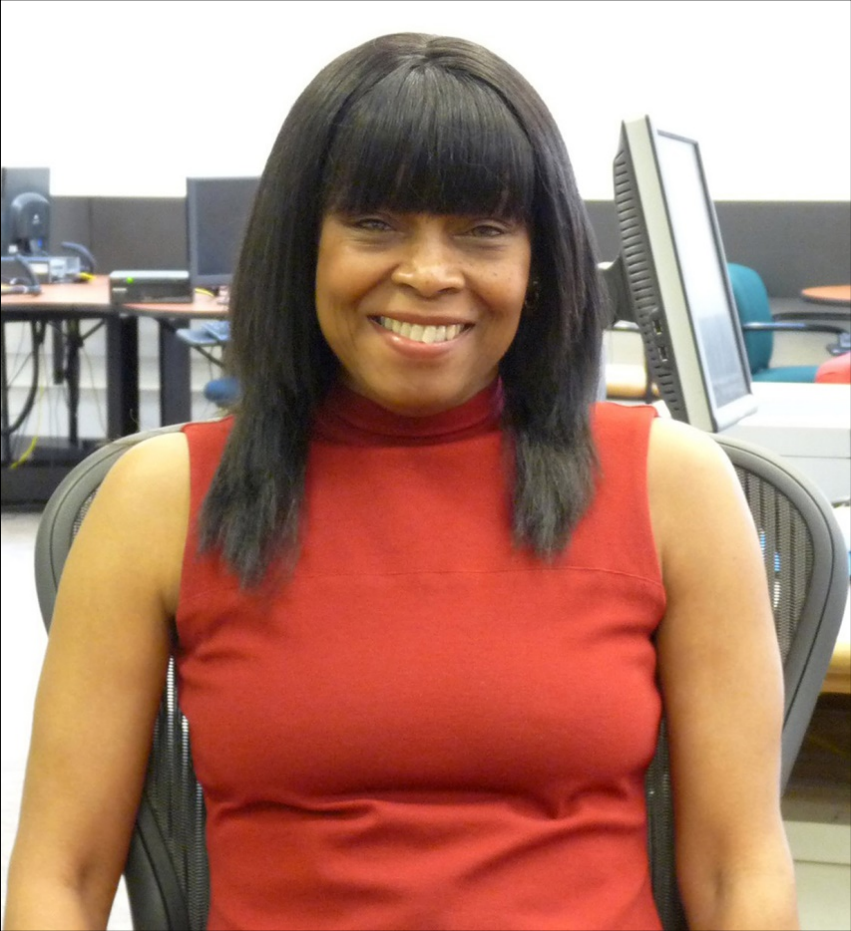


Beverly’s MLA service has been typified by a series of courageous moments where she was the first to take on certain roles, demonstrating her boldness. Beverly’s boldness motivated her to chart a path or take on roles in MLA that no one had done before her. For example, in 2000, she became the first African American to be appointed to the role of editor of the *MLA News,* a role she held for six years. Under her leadership, the newsletter introduced several new columns. As editor, she was instrumental in launching the online edition of the *MLA News.* She was also the first chair of the Professional Recruitment and Retention Committee (formerly, an ad hoc committee) after it was established as a standing MLA committee in 2006. Under her leadership, the committee worked closely with the MLA Placement Center to establish the Resume Clinic, which continues to be a value-added member resource today.

As Beverly takes on the role of president of MLA, she will take on another first. Anyone who has taken on the role of being “the first” knows all too well the significant pressure and responsibility that comes with that role. All eyes are on you as people look to you to set the example. That role often opens the person up to unfair criticism and public scrutiny. That withstanding, I believe she is well suited to be first. She has taken each of the aforementioned roles with sass, elegance, pizzazz, and humor, all of which will serve her well during her presidential year.

Beverly is also an active member of the Mid-Atlantic Chapter (MAC) of MLA, where she has served the association in a variety of roles. Most notably, she served as treasurer from 1997–1999 and as chapter chair in 2003–2004. Her other roles included chairing the Financial Planning Task Force in 1999–2000, the Publications Committee in 1998–1999, Strategic Planning Task Force in 2004–2005, and the Communications Committee in 2014–2015. She also served as editor of *MAC Messages,* the association’s newsletter in 1995–1998. Her MAC colleagues recognized her service and contributions to the chapter by awarding her the Marguerite Abel Service Recognition Award in 1998 and the MAC Librarian of the Year Award (now the MAC Award for Professional Excellence by a Health Sciences Librarian) in 2005.

Beverly is a strong proponent and supporter of diversity and inclusion in librarianship; so much so that her efforts in this area have been acknowledged locally by the North Carolina Library Association’s Round Table for Ethnic Minority Concerns when the group awarded her its Roadbuilder’s Award for Special Librarianship. Her passion for diversity and inclusion work was evident when she delivered her inaugural presidential address at MLA ’18 and stated:

No matter what race we are, what color we are, what ethnicity we are, what gender we have, or whether we have physical issues—we are all information professionals, with a common goal, and that is “to be an association of the most visible, valued, and trusted health information experts.” Diversity drives excellence and makes us smarter, especially when we welcome it into our lives, our libraries, and our profession.

She is currently channeling her passion for diversity and inclusion work as the coeditor of a forthcoming book, *Diversity and Inclusion in Libraries: A Changing Facet of Librarianship,* to be published in 2019.

From the moment that I met Beverly, I knew that she was someone special. Her welcoming and warm nature made me feel at ease instantly. I knew, from that point, we would become fast friends. She made me feel like I mattered.

In fall 2000, I relocated to Raleigh, North Carolina, from my hometown in Norfolk, Virginia, to pursue a master’s in library science from NCCU in Durham. I was eager to begin my studies, anxious to learn as much about being a health sciences librarian as possible, and excited about the possibility of meeting librarians from whom I could learn the ropes. Prior to enrolling in library and information science (LIS) school, I was cautioned that African Americans were underrepresented in librarianship. NCCU’s School of Library and Information Sciences (SLIS) is the only program of its type at a historically black college or university (HBCU). I arrived on campus in spring 2001, excited to begin my coursework and interact with fellow classmates, especially people of color (POC) who were pursuing the same degree. Surprisingly, SLIS’s student body population reflected the demographic of the profession. Even at an HBCU, I was a minority in the school.

During my second semester in the program, I attended a continuing education course offered by the Association of North Carolina Health and Science Libraries (ANCHASL), “Grant Writing for Librarians.” It was here that I met SLIS alumna Beverly Murphy. I had the opportunity to work with her throughout the day and share with her my career aspirations. I appreciated her candor and genuine demeanor. As the workshop was coming to an end, she invited me to visit her library, and I remember telling her, “You are stuck with me now.” She responded, “We are stuck with each other,” and that we have been.

One of my favorite quotes from Mary Church Terrell captures that nature of my relationship with Beverly nicely. In a February 1898 speech to the National American Women’s Suffrage Association, Terrell told her audience that we must continue “lifting as we climb, onward and upward we go, struggling and striving, and hoping that the buds and blossoms of our desires will burst into glorious fruition ere long” [1]. This quote resonates with me because it suggests that “despite our own hardships, when you succeed, you should use that position of power, of privilege, of success to ‘lift’ other women up with you” [2].

As I reflect on the impact that Beverly has had on my career, she has done exactly what Terrell suggested in that famous quote: she has lifted me as she climbed. Since 2001, Beverly has served as a role model, a mentor, a trusted advisor, and, most importantly, my friend. She assisted me with establishing a record of service in MLA and MAC of MLA, as I learned to navigate MLA, and introducing me to the membership of the African American Medical Librarians Alliance (AAMLA) Special Interest Group (SIG) during my inaugural years in the profession. She has inspired me to bring my very best self to everything that I do as a librarian.

Over the years, I have grown to admire Beverly’s tenacious spirit, authenticity, fearlessness, and her passion for librarianship. As a librarian of color, she was what I needed early in my profession, as her mere presence ensured that I had a librarian of color whom I could look up to and from whom I could seek advice. More importantly, the impact of my interactions with Beverly during my early years in the profession affirmed the idea that representation matters. Representation in the profession is just as important today as it was in 2001. Librarianship is a profession that is largely white and female and can be challenging for POC when they are new to health sciences librarianship. It matters that POC see themselves reflected in our libraries especially when it comes to the staff who work at the library. I have learned many lessons from Beverly, none more important than the responsibility to support librarians coming up after me. She did this for me and challenged me to pay it forward by lifting as I climbed the ranks.

Beverly’s commitment to sharing her expertise and knowledge with colleagues is apparent. She has mentored and sponsored countless colleagues in the profession for years. James Dale Prince, AHIP, director, Libraries, Louisiana State University Health Sciences Center, noted that he has learned much about Beverly’s character in the seventeen years that he has known her. He shared that Beverly is inclusive and empathetic. She approached him at his first library conference because she knew he was new. She wanted to make sure he felt welcome. She let him know that resources (meaning her) were available should he need them.

He also described Beverly as stubborn and passionate, sharing that she was adamant with the 2019 National Planning Committee that there would be no exclusive events centered around her as president, arguing that invitation-only events make people feel left out. “I’ve already talked to Kevin [Baliozian] about this, and it’s not happening,” she told Prince. Prince shared a sentiment that Beverly’s closest colleagues know all too well:

Beverly is opinionated and infuriating, but that’s because she’s often right. She’s joyful. She’s quick to laugh and to make others laugh, a great attribute when you are on as many committees as Beverly. And finally, and this may be her organizing principle, Beverly thinks other people are important. This informs her welcoming nature and her passion for inclusion. And it means she’s going to call you on it when your plan ignores the feelings of others.

Meredith Solomon, AHIP, outreach officer at Harvard University’s Countway Library, who has been friends with Beverly since 2006, described Beverly as a dynamic, results oriented, energetic, shoot-from-the-hip woman. Solomon is elated that Beverly was elected MLA’s 2018/19 president and its first African American president in the history of MLA. Beverly has a vision but also knows that membership input and involvement in our forward momentum is integral for MLA to become greater and stronger: “I look forward to seeing how she and the membership accomplish our goals together.”

Beverly’s impact has been felt throughout health sciences librarianship by librarians at all levels. When asked about what impact she has had on them and what her presidency means to them, her MLA colleagues had this to say:

- Virginia (Ginger) Carden, AHIP, administrative research librarian at the Duke Medical Center Library shared that Beverly has been a colleague and friend for more than thirty years. She writes, “It has been a privilege to know and work with Beverly. She is open to new people and new skills. She is someone you can turn to when you have an issue, be it work or personal as she will listen and offer to help anyway she can. MLA is lucky to have her as our president as she is cognizant, aware, and inclusive. Just what we need as we move forward in these challenging time.”
- Brenda Faye Green, associate vice president, Institutional Technology, and library director at Meharry Medical College, shared that throughout her career, “Beverly has remained true to her métier—passionate about our profession, energetic, loyal to friends and colleagues alike, and always ready to meet next generation, early career, and librarians of color.” She notes, “During Beverly’s presidency, MLA members will see inclusivity, frank and transparent communication, and an appreciation and respect for MLA’s staff, elected and appointed officers, and stakeholders who have sustained MLA’s foundation over the years. Beverly’s inaugural address began with a dialogue and I believe she will continue that dialogue, with our community of practice, throughout her time as president.”
- PJ Grier, associate director, Library and Educational Information Systems, OJ Snyder Memorial Library, Philadelphia College of Osteopathic Medicine, expects Beverly’s presidency to be transparent and open, by inspiring MLA members to reach beyond our boxes and silos. PJ has worked “worked closely with Beverly on several projects including coauthorship in a peer-reviewed journal and on various committees in support of Regional Medical Library outreach initiatives. He finds Beverly clear, concise in thought and action, and supportive of people and agencies in which she maintains professional relationships.
- Nisha Mody, health and life sciences librarian at the University of California–Los Angeles (UCLA) and coconvener of MLA’s New Member SIG, shared that when she met Beverly, she saw someone who was a go-getter, positive, and someone who believes in community and collaboration. She told me, “I saw someone that was willing to engage with others with love and compassion, someone that understands how boundaries can be pushed.”
- Terri Ottosen, AHIP, community engagement and health literacy librarian at the University of North Carolina’s Health Sciences Library, said of Beverly, “I can’t recall the exact circumstance under which I met Beverly Murphy the first time, as it seems that I’ve known her all my life. There are very few people I’ve instantly connected with and didn’t feel that I had to measure my words before speaking. Quite simply, Bev gets me and impressively, she gets people and goes out of her way to make others feel comfortable. I know she understands so much about what MLA members want, having served this national organization as well as local and regional professional organizations with vitality, for many years. I’m excited for her to lead us, making an impact by listening, responding to our concerns as a profession, and envisioning our future. At her core, she gets it.”
- Heather N. Holmes, AHIP, associate director of libraries at the Medical University of South Carolina, met Beverly at the 2014 MAC meeting in Alexandria, Virginia: “Of course I knew her name and reputation, so I was very excited to finally get to be introduced to her. She greeted me like we were long, lost friends and immediately I knew everything that I’d heard about her was true. I’ve been fortunate to work with her more closely over the past year while she served as President-Elect, and I chaired the Research and Evidence-Based Practice Curriculum Committee. I’ve spoken with her when I’ve needed advice, guidance, and when I’ve needed someone to bring me back to reality. Beverly has the gift of making you feel like you’re the most important person in the world while you’re talking with her. She’s kind and respectful, and she listens to what you’re saying. I’m lucky to call her a colleague, and even more lucky to call her a friend. MLA is fortunate to have someone like her at the helm for this coming year, and I am excited to see where she leads the organization.”

Overall, there is collective excitement and enthusiasm about Beverly’s MLA presidency. When I think about what she means to me, her MLA colleagues, and the profession, this quote from Maya Angelou sums it up best: “I’ve learned that people will forget what you said, people will forget what you did, but people will never forget how you made them feel” [3]. Beverly has made individuals throughout the association feel like they matter by being a strong advocate for MLA members at all levels in the association and at all stages of their careers as well as embracing the unique talents, skills, and diversity that people bring to the association.

Personally, I am proud of her for all that she has done and will continue to do for MLA. I am grateful that she answered the call to serve the association in this fashion. I appreciate the bravery that she has shown in stepping into the role of president. Most importantly, I thank her for recognizing that accepting the role of president was not just about her, but also about being the change that we all needed to see in our association. Finally, great leaders inspire others to action, so I am prepared to do the necessary work to ensure that Beverly’s presidential year is positive, productive, and impactful for her and the association. I invite each of you to join our president on this journey to just “Be”: Be Bold, Be Brave, Be Brilliant.
